# Memory

**Published:** 1883-12

**Authors:** 


### MEMORY.

A man's memory is like his stomach. To do its best work it must have good treatment. It must neither be neglected nor overloaded. It can easily be so abused by neglect, or by irregular and unsystematic employment, as to become chiefly a cause of annoyance and discomfort; or, again, it can be so overworked and heavily taxed that it becomes practically the chief organ or agent of the entire system, every other portion dwindling in its comparison. The latter course is the great danger of those who value the help of a tenacious memory.

Both memory and stomach are valuable, not in proportion to the burdens they can carry, but in proportion to their training for their part in the work of the system as a whole; and either of them is made effective as much by what is kept from it as by what is packed into it.

